# Healthcare resource utilization and medical costs in patients with terminal cancer during best supportive care

**DOI:** 10.1371/journal.pone.0269565

**Published:** 2022-06-03

**Authors:** Dong-Won Kang, Yoon-Bo Shim, Eui-Kyung Lee, Mi-Hai Park

**Affiliations:** School of Pharmacy, Sungkyunkwan University, Suwon, Gyeonggi-do, South Korea; Chang Gung Memorial Hospital and Chang Gung University, Taoyuan, Taiwan, TAIWAN

## Abstract

Patients with terminal cancer have different physical symptoms, prognoses, emotional distress, and end-of-life care plans from those receiving aggressive chemotherapy; few studies have assessed healthcare resource use in these patients. Therefore, this study aimed to assess healthcare resource utilization and medical costs incurred during best supportive care after the last anticancer drug treatment in patients with terminal cancer. This retrospective observational study was conducted using national sample cohort data from the National Health Insurance Service in South Korea. Only patients with cancer who were treated with the last anticancer drugs from January 1, 2006, to June 30, 2015, were included in the study. The period of best supportive care was defined as the time from the date of use of the last anticancer drug to death. Healthcare resource utilization and medical costs were estimated during the best supportive care. A generalized linear model with a log-link function and gamma distribution was used to evaluate the impact of demographic and healthcare utilization factors on total medical costs. Among the 2,480 patients in the study, 93.9% were hospitalized, and hospitalization days (30.8 days) accounted for 39.7% of the surviving period (77.5 days). The proportions of intensive care unit admissions and emergency department visits were 15.8% and 18.9%, respectively. The average total medical cost per patient was $6,310, with the inpatient cost ($5,705) being approximately 9.4 times higher than the outpatient cost ($605). The length of hospitalization had the greatest impact on the total medical costs. Pancreatic cancer had the highest proportion of patients who were hospitalized (97.4%) and the highest medical cost ($7,702). Hospital-based resources were utilized by most patients with terminal cancer, and hospitalization was a major driver of the total medical cost. An alternative system for hospitalization should be developed to support patients with terminal cancer, both clinically and financially.

## Introduction

Approximately 18.1 million new cases of cancer were reported globally in 2018, and 9.6 million people died of cancer that year [1, 2]. As patients approach death or there are no more treatments for their cancer, the need for the best supportive care increases. The best supportive care is to prevent or treat symptoms in patients with terminal cancer who can no longer be treated with chemotherapy. It is estimated that the number of people dying of cancer who will require the best supportive care in 2060 will be 16.3 million globally, nearly double the total in 2016 [3, 4].

The economic burden of patients with terminal cancer rises sharply as they approach death [5, 6]. To reduce the economic burden and determine how best to support them, it is necessary to understand the healthcare resources they use and where medical costs come from. In addition, terminally ill patients for whom there are no further treatments differ from those receiving aggressive chemotherapy in terms of physical symptoms, prognosis, emotional distress, and end-of-life care planning [7, 8]. They seek a comfortable death and prefer symptom relief over aggressive and non-beneficial treatments [9]. Since healthcare resource utilization and medical costs may also differ due to these different characteristics, it is necessary to distinguish incurable patients from patients receiving active treatment to identify the best supportive care.

A previous study conducted in Australia reported the medical resource use and medical costs of patients with terminal cancer during the end-of-life period [10]. Another study performed in Belgium showed the healthcare resource utilization and medical costs of patients with cancer in the six months before death [11]. However, as these studies included patients with cancer who were receiving active treatment, such as chemotherapy, they could not show the exact state of patients with untreated conditions. Therefore, this study aimed to assess healthcare resource utilization and medical costs during best supportive care in patients with terminal cancer using the National Health Insurance claims data in South Korea.

## Methods

### Data sources

The national sample cohort data from the National Health Insurance Service (NHIS-NSC) for the period between January 1, 2002 and December 31, 2015 were used. The NHIS-NSC provides representative data extracted from the Korean population and contains information on approximately one million individuals. These one million individuals account for 2.2% of the total eligible population. The database contains data on demographic characteristics such as sex, age, and month of death; and data on healthcare resource utilization such as disease diagnoses, medical procedures, costs, and medication use [12]. All patient data were anonymized before access. This study was approved by the Institutional Review Board of Sungkyunkwan University on January 16, 2020 (IRB: 2020-01-009). Data access was approved from June 2020 to September 2021 by the NHIS (data number: NHIS-2020-2-083).

### Study population

Cancers with globally high mortality rates, including lung, liver, colon, gastric, pancreatic, gallbladder, and biliary cancers, were examined in this study [2]. The cancer diagnosis was coded using the International Classification of Diseases, 10th Revision (lung cancer: C33, C34; liver cancer: C22; colon cancer: C18, C19, C20, C21; gastric cancer: C16; pancreatic cancer: C25; gallbladder and biliary cancer: C23, C24).

Patients with terminal cancer were defined as those who died within six months of the last anticancer treatment [13, 14]. The anticancer drugs (Anatomical Therapeutic Chemical [ATC] code, L01) were selected based on their ATC classification. The index date was defined as the date of use of the last anticancer drug and the best supportive care period was defined as the number of days from the index date to death. Only patients with an index date between January 1, 2006 and June 30, 2015 were included to ensure a follow-up period of at least six months. The pre-index period was defined as six months before the index date to assess the baseline characteristics of the patients. To verify the diagnosis of cancer, only patients with at least two independent claims data associated with each single cancer during the pre-index period were included. Patients with claims associated with two or more different types of cancer were excluded to reduce the bias of medical costs, and those without claims after the index date or who died at the index date were excluded. Eligible patients were followed up from the index date until death.

### Study measures

Demographic characteristics, healthcare resource utilization, and medical costs were evaluated in this study. They were assessed according to cancer type owing to differences in clinical features and treatment patterns. Age and sex were identified on the index date, and the Charlson Comorbidity Index (CCI) was calculated within six months before the index date [15, 16]. Healthcare resource utilization included hospitalization, outpatient visits, emergency department (ED) visits, and intensive care unit (ICU) admission. These variables were selected based on previous studies [10, 11, 17, 18]. Hospitalization periods and the number of outpatient visits can show how many medical resources patients typically use. The number of hospitalizations in the ICU and the rate of ED visits indicate how much the patients require urgent medical support.

The best supportive care costs were calculated for each patient and categorized as either inpatient or outpatient costs. Only total healthcare costs, including co-payment by patients and coverage by the National Health Insurance, were considered in this study, and the non-reimbursed costs were excluded. Inpatient and outpatient costs included the cost of admission (for inpatients only), administration and injection, medication, procedures, lab testing, imaging, and other aspects of the care provided. The cost proportion of each item was estimated to identify the items affecting inpatient and outpatient costs. To adjust for the impact of different survival times for cancer types after the last anticancer drug, per patient per month (PPPM) costs were also estimated by dividing the total medical costs of each patient by the period after the index date until death.

### Statistical analysis

Categorical variables are presented as numbers with proportions, and continuous variables are presented as medians or means with standard deviations for each cancer type. To compare the characteristics, healthcare resource utilization, and medical costs between cancer types, categorical variables were compared using the chi-squared test, and continuous variables were compared using Welch’s analysis of variance [19]. A generalized linear model with a log-link function and gamma distribution was used to evaluate the impact of demographic and healthcare utilization factors on total medical costs. Demographic factors (age group, sex, CCI, and the length of the period from the last anticancer drug administration to death) and healthcare resource utilization (hospitalization, ICU admission, outpatient visits, and ED visits) were included in the model as covariates. There was no multicollinearity when the variance inflation factor was less than 2.5. All analyses were performed using SAS version 9.4 (SAS Institute, Cary, NC, USA).

## Results

**Fig 1:**
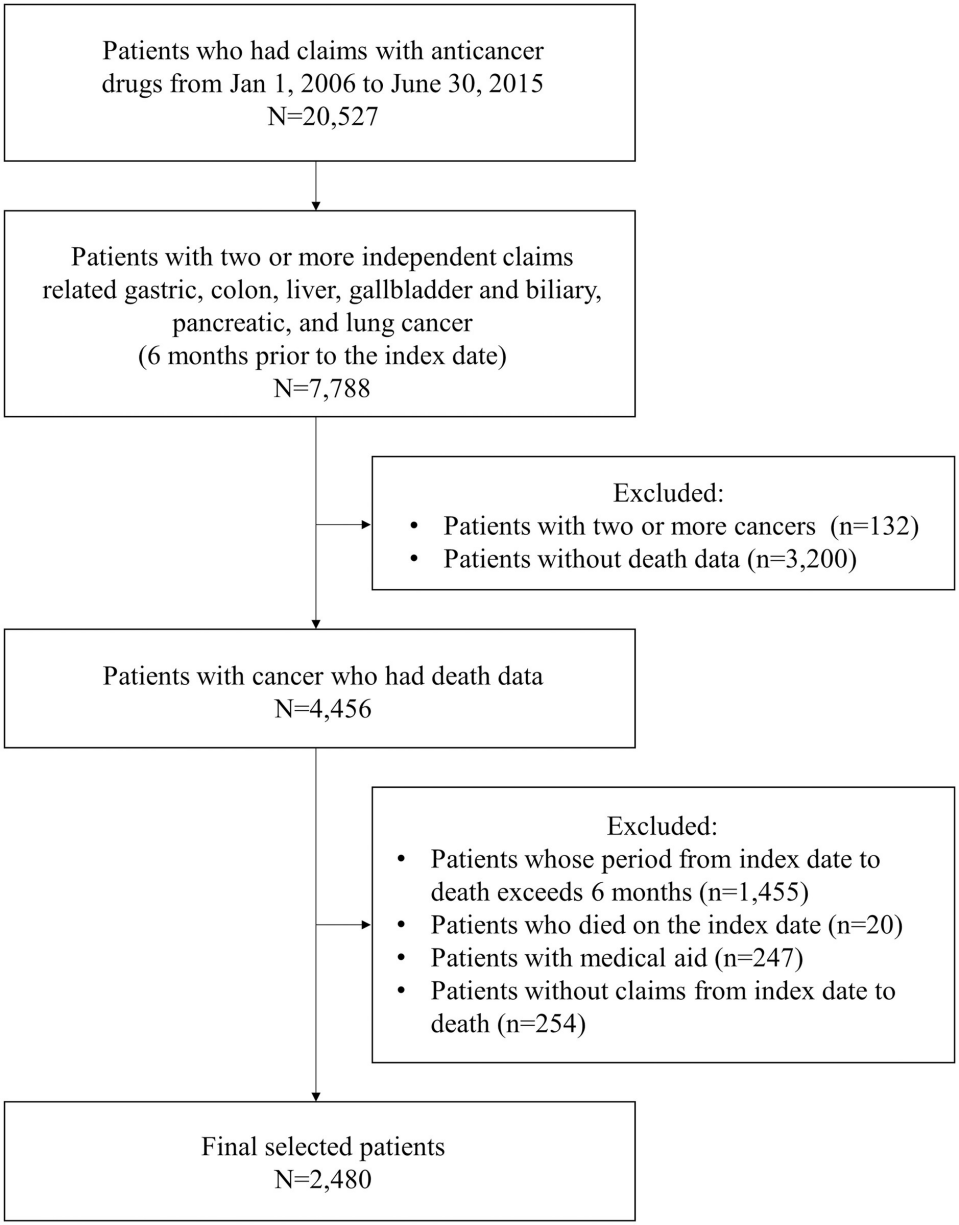
Flow chart of patient selection.

Among the patients who had claims for anticancer drugs from January 1, 2006 to June 30, 2015, a total of 2,480 patients were selected for this study (Fig 1). The mean age of the patients was 63.3 years. The patient with lung cancer was the oldest (66.0 years), and the patient with liver cancer was the youngest (60.0 years). The highest proportion of male patients was in the group of patients with liver cancer (82.2%), followed by those with lung cancer (78.5%). The average period until death after the last anticancer drug administration was 77.5 days in all patients, with the longest in the patients with gallbladder and biliary cancer (89.1 days) and the shortest in patients with lung cancer (70.9 days; Table 1).

**Table 1:** Demographic characteristics of patients with terminal cancer.

	All (N = 2,480)	Type of cancer						P value
		Lung cancer (n = 782)	Liver cancer (n = 590)	Gastric cancer (n = 518)	Colon cancer (n = 315)	Pancreatic cancer (n = 194)	Gallbladder and biliary cancer (n = 81)	
Age group, n (%)								
Mean (SD)	63.3 (11.1)	66.0 (9.7)	60.0 (10.9)	61.5 (12.6)	64.8 (11.1)	63.5 (9.7)	65.8 (9.1)	<0.001
<50	290 (11.7)	48 (6.1)	97 (16.4)	93 (18.0)	32 (10.2)	18 (9.3)	2 (2.5)	<0.001
50–64	961 (38.8)	253 (32.4)	282 (47.8)	204 (39.4)	104 (33.0)	84 (43.3)	34 (42.0)	
65–79	1,123 (45.3)	446 (57.0)	197 (33.4)	191 (36.9)	164 (52.1)	86 (44.3)	39 (48.2)	
≥80	106 (4.3)	35 (4.5)	14 (2.4)	30 (5.8)	15 (4.8)	6 (3.1)	6 (7.4)	
Sex, n (%)								
Male	1,810 (73.0)	614 (78.5)	485 (82.2)	344 (66.4)	199 (63.2)	123 (63.4)	45 (55.6)	<0.001
Female	670 (27.0)	168 (21.5)	105 (17.8)	174 (33.6)	116 (36.8)	71 (36.6)	36 (44.4)	
Charlson Comorbidity Index, n (%)								
Mean (SD)	7.7 (3.3)	7.0 (3.4)	8.7 (3.5)	7.6 (2.9)	7.9 (2.9)	7.9 (3.4)	7.4 (3.3)	<0.001
<9	1,452 (58.6)	449 (57.4)	310 (52.5)	339 (65.4)	186 (59.1)	116 (59.8)	52 (64.2)	0.001
≥9	1,028 (41.5)	333 (42.6)	280 (47.5)	179 (34.6)	129 (41.0)	78 (40.2)	29 (35.8)	
Type of hospital, n (%)								
Tertiary hospital	1552 (62.6)	303 (58.5)	185 (58.7)	405 (68.6)	47 (58.0)	134 (69.1)	478 (61.1)	0.002
General hospital	713 (28.8)	158 (30.5)	104 (33.0)	149 (25.3)	31 (38.3)	43 (22.2)	228 (29.2)	
Hospital	91 (3.7)	23 (4.4)	7 (2.2)	17 (2.9)	2 (2.5)	9 (4.6)	33 (4.2)	
Clinic	57 (2.3)	15 (2.9)	13 (4.1)	11 (1.9)	0 (0)	5 (2.6)	13 (1.7)	
Others	67 (2.7)	19 (3.7)	6 (1.9)	8 (1.4)	1 (1.2)	3 (1.6)	30 (3.8)	
Period from the last anticancer drug administration to death, n (%)								
Mean days (SD)	77.5 (44.3)	70.9 (45.3)	81.9 (43.0)	75.1 (41.0)	84.0 (49.6)	81.5 (40.1)	89.1 (44.1)	<0.001
<1 month	342 (13.8)	152 (19.4)	65 (11.0)	62 (12.0)	43 (13.7)	13 (6.7)	7 (8.6)	<0.001
1 month to 2 months	670 (27.0)	227 (29.0)	138 (23.4)	151 (29.2)	80 (25.4)	58 (30.0)	16 (19.8)	
2 months to 3 months	548 (22.1)	159 (20.3)	141 (23.9)	130 (25.1)	56 (17.8)	41 (21.1)	21 (25.9)	
3 months to 4 months	420 (16.9)	100 (12.8)	116 (19.7)	99 (19.1)	50 (15.9)	37 (19.1)	18 (22.2)	
4 months to 5 months	280 (11.3)	82 (10.5)	73 (12.4)	38 (7.3)	45 (14.3)	33 (17.0)	9 (11.1)	
5 months to 6 months	220 (8.9)	62 (7.9)	57 (9.7)	38 (7.3)	41 (13.0)	12 (6.2)	10 (12.4)	

Abbreviations: SD, standard deviation.

During best supportive care, 93.9% of the patients were hospitalized and 80.1% had at least one outpatient visit. The average number of hospitalizations was 1.6, with an average of 30.8 hospitalization days, accounting for 39.7% of the best supportive care period. The proportions of ICU admissions and ED visits were 15.8% and 18.9%, respectively. Among all cancers, inpatient resource use was the most common for pancreatic cancer, with 97.4% of patients hospitalized once, and an average hospitalization duration of 38.1 days (Table 2). For medications used during best supportive care, patients were prescribed opioid and non-opioid analgesics more than other medications (S1 Fig).

**Table 2:** Healthcare resource utilization of patients with terminal cancer.

	All (N = 2,480)	Type of cancer						P value
		Lung cancer (n = 782)	Liver cancer (n = 590)	Gastric cancer (n = 518)	Colon cancer (n = 315)	Pancreatic cancer (n = 194)	Gallbladder and biliary cancer (n = 81)	
**Resource utilization of inpatients**								
Number of patients^a^, n (%)	2,329 (93.9)	723 (92.5)	564 (95.6)	494 (95.4)	283 (89.8)	189 (97.4)	76 (93.8)	<0.001
Number of hospitalizations per patient, mean (SD)	1.6 (1.1)	1.5 (1.0)	1.8 (1.2)	1.6 (1.1)	1.5 (1.0)	1.8 (1.2)	2.0 (1.5)	<0.001
Hospitalization days per patient, mean (SD)	30.8 (28.1)	28.2 (28.2)	28.8 (26.1)	34.1 (28.3)	29.8 (29.8)	38.1 (29.3)	35.2 (27.1)	<0.001
Hospitalization days compared with survival days (%)	39.7	39.8	35.2	45.4	35.5	46.7	39.5	
Number of patients hospitalized in ICU^a^, n (%)	393 (15.8)	155 (19.8)	97 (16.4)	60 (11.6)	47 (14.9)	23 (11.9)	11 (13.6)	0.002
**Resource utilization of outpatients**								
Number of patients^a^, n (%)	1,987 (80.1)	594 (76.0)	502 (85.1)	396 (76.5)	263 (83.5)	162 (83.5)	70 (86.4)	<0.001
Number of outpatient visits per patient, mean (SD)	3.9 (5.4)	3.6 (5.5)	4.3 (5.4)	3.4 (4.8)	4.3 (5.1)	4.5 (6.3)	4.5 (5.7)	0.008
**ED visits**								
Number of patients^a^, n (%)	469 (18.9)	132 (16.9)	119 (20.2)	102 (19.7)	55 (17.5)	45 (23.2)	16 (19.8)	0.335
Rate of ED visits per month^b^, mean (SD)	0.16 (0.81)	0.18 (0.80)	0.14 (0.44)	0.13 (0.37)	0.21 (1.71)	0.14 (0.35)	0.10 (0.25)	0.440

Abbreviations: ED, emergency department; ICU, intensive care unit; SD, standard deviation.

^a^The percentage was calculated by dividing the number of patients by the total number of patients with each cancer type.

^b^The rate of ED visits per month was calculated by dividing the number of ED visits by the total survival period.

The average total medical cost per patient was $6,310, and the PPPM cost was $3,072. The inpatient cost ($5,705) was approximately 9.4 times higher than the outpatient cost ($605). The total medical cost for patients with pancreatic cancer was the highest, with an average of $7,702, and the medical cost for patients with gallbladder and biliary cancers was the second-highest, with an average of $7,378. Similarly, the average cost of inpatient care was the highest for pancreatic cancer ($7,131) and the second-highest for gallbladder and biliary cancer ($6,737). The PPPM costs for lung cancer were $3,642, $3,376, and $266 in the total patient, inpatient, and outpatient groups, respectively; which were higher than those for other cancers (Table 3).

**Table 3:** Medical costs accrued by patients with cancer during best supportive care.

	All (N = 2,480)	Type of cancer						P value
		Lung cancer (n = 782)	Liver cancer (n = 590)	Gastric cancer (n = 518)	Colon cancer (n = 315)	Pancreatic cancer (n = 194)	Gallbladder and biliary cancer (n = 81)	
**Costs per patient during best supportive care**								
Total medical cost ($US)								
Mean (SD)	6,310 (6,397)	5,790 (5,097)	6,712 (8,093)	6,304 (5,838)	5,724 (5,811)	7,702 (7,475)	7,378 (5,793)	0.001
Median	4,819	4,435	5,091	4,905	4,041	5,325	5,874	
Inpatient cost ($US)								
Mean (SD)	5,705 (6,291)	5,156 (4,923)	5,962 (7,964)	5,899 (5,818)	5,119 (5,737)	7,131 (7,374)	6,737 (5,702)	<0.001
Median	4,173	3,869	4,270	4,567	3,439	5,011	5,428	
Outpatient cost ($US)								
Mean (SD)	605 (1,062)	634 (1,186)	750 (1,298)	405 (666)	605 (911)	571 (712)	641 (1,013)	<0.001
Median	273	223	414	172	312	281	311	
**PPPM costs during best supportive care**								
Total medical cost ($US)								
Mean (SD)	3,072 (4,668)	3,642 (7,099)	2,690 (2,390)	2,983 (3,217)	2,571 (2,665)	3,156 (3,908)	2,675 (2,003)	0.003
Median	2,182	2,263	2,197	2,131	1,852	2,496	2,102	
Inpatient cost ($US)								
Mean (SD)	2,839 (4,679)	3,376 (7,114)	2,424 (2,385)	2,824 (3,232)	2,328 (2,678)	2,968 (3,923)	2,467 (2,057)	0.001
Median	1,947	1,965	1,937	1,988	1,502	2,310	1,798	
Outpatient cost ($US)								
Mean (SD)	233 (452)	266 (551)	266 (453)	159 (268)	243 (542)	188 (196)	208 (286)	<0.001
Median	120	114	157	79	121	120	113	

Abbreviations: SD, standard deviation; PPPM, per patient per month.

US$1 = 1172.50 won.

Hospitalization days had the greatest impact on total medical costs for all cancers, according to the generalized linear model (Table 4). The total medical cost of hospitalized patients was higher than that of patients who were not hospitalized, and the total medical cost tended to sharply increase with longer hospitalization stays for all cancers. A longer period between the last anticancer drug treatment and death was also related to higher total medical costs in general. On the other hand, most demographic factors did not significantly affect total medical costs during best supportive care.

**Table 4:** Generalized linear model analysis evaluating the impact of demographic and healthcare utilization factors on medical costs during best supportive care.

	Lung cancer	Liver cancer	Gastric cancer	Colon cancer	Pancreatic cancer	Gallbladder and biliary cancer
	OR (95% CI)	OR (95% CI)	OR (95% CI)	OR (95% CI)	OR (95% CI)	OR (95% CI)
Age group						
<50 (ref.)	-	-	-	-	-	-
50–64	1.05 (0.87–1.28)	0.96 (0.84–1.10)	0.90 (0.77–1.05)	0.78 (0.59–1.02)	1.08 (0.81–1.43)	0.46 (0.18–1.20)
65–80	1.04 (0.86–1.26)	0.92 (0.80–1.06)	0.71 (0.61–0.83)	0.73 (0.57–0.95)	0.87 (0.66–1.16)	0.41 (0.16–1.05)
≥80	0.86 (0.66–1.13)	0.86 (0.61–1.20)	0.80 (0.63–1.03)	0.66 (0.44–1.01)	0.62 (0.36–1.04)	0.35 (0.12–1.01)
Sex						
Male (ref.)	-	-	-	-	-	-
Female	0.91 (0.82–1.02)	0.92 (0.81–1.04)	0.98 (0.87–1.10)	0.84 (0.72–0.98)	1.05 (0.88–1.24)	0.95 (0.70–1.28)
Period from the last anticancer drug administration to death						
<1 month (ref.)	-	-	-	-	-	-
1 month to 2 months	1.22 (1.07–1.40)	1.51 (1.26–1.81)	1.08 (0.90–1.31)	1.10 (0.86–1.42)	1.04 (0.74–1.48)	2.07 (1.18–3.66)
2 months to 3 months	1.30 (1.12–1.51)	1.58 (1.31–1.91)	1.28 (1.04–1.56)	1.39 (1.05–1.84)	1.01 (0.69–1.48)	2.25 (1.24–4.10)
3 months to 4 months	1.54 (1.29–1.82)	1.92 (1.57–2.35)	1.49 (1.19–1.86)	1.16 (0.86–1.58)	1.03 (0.70–1.54)	2.86 (1.57–5.20)
4 months to 5 months	1.92 (1.59–2.31)	1.74 (1.40–2.17)	1.46 (1.11–1.91)	1.42 (1.05–1.93)	1.53 (1.02–2.30)	3.20 (1.56–6.55)
5 months to 6 months	2.16 (1.78, 2.63)	2.54 (2.01, 3.21)	1.60 (1.22, 2.08)	1.83 (1.34, 2.52)	1.31 (0.81, 2.11)	1.73 (0.85, 3.51)
Hospitalization days						
No hospitalization (ref.)	-	-	-	-	-	-
1–20 days	5.65 (4.74–6.74)	8.10 (6.36–10.33)	7.33 (5.64–9.52)	6.18 (4.60–8.32)	6.57 (3.92–11.02)	1.70 (0.85–3.42)
21–40 days	10.50 (8.70–12.66)	14.92 (11.61–19.17)	15.01 (11.49–19.62)	11.66 (8.55–15.89)	12.71 (7.54–21.42)	3.95 (2.03–7.69)
>40 days	15.33 (12.67–18.56)	24.50 (18.82–31.89)	22.95 (17.43–30.21)	18.97 (13.72–26.22)	20.29 (11.82–34.82)	7.48 (3.90–14.34)
ICU hospitalization						
Yes (ref. No)	2.02 (1.81–2.26)	1.41 (1.24–1.61)	1.94 (1.65–2.29)	1.72 (1.39–2.14)	1.52 (1.17–1.97)	1.37 (0.90–2.09)
Outpatient visits						
Yes (ref. No)	0.95 (0.85–1.06)	0.97 (0.83–1.12)	0.92 (0.80–1.05)	0.92 (0.73–1.15)	0.88 (0.69–1.11)	1.08 (0.70–1.68)
ED visits						
Yes (ref. No)	1.05 (0.93–1.18)	1.11 (0.99–1.26)	1.13 (0.99–1.29)	1.20 (0.97–1.47)	1.20 (0.98–1.46)	0.89 (0.60–1.32)

Abbreviations: OR, odds ratio; CI, confidence interval; ICU, intensive care unit; ED, emergency department.

The results were adjusted for age, sex, Charlson comorbidity index, period from the last anticancer drug administration to death, hospitalization days, ICU hospitalizations, outpatient visits, and ED visits.

The variance inflation factor was less than 2.5 for all covariates.

**Fig 2:**
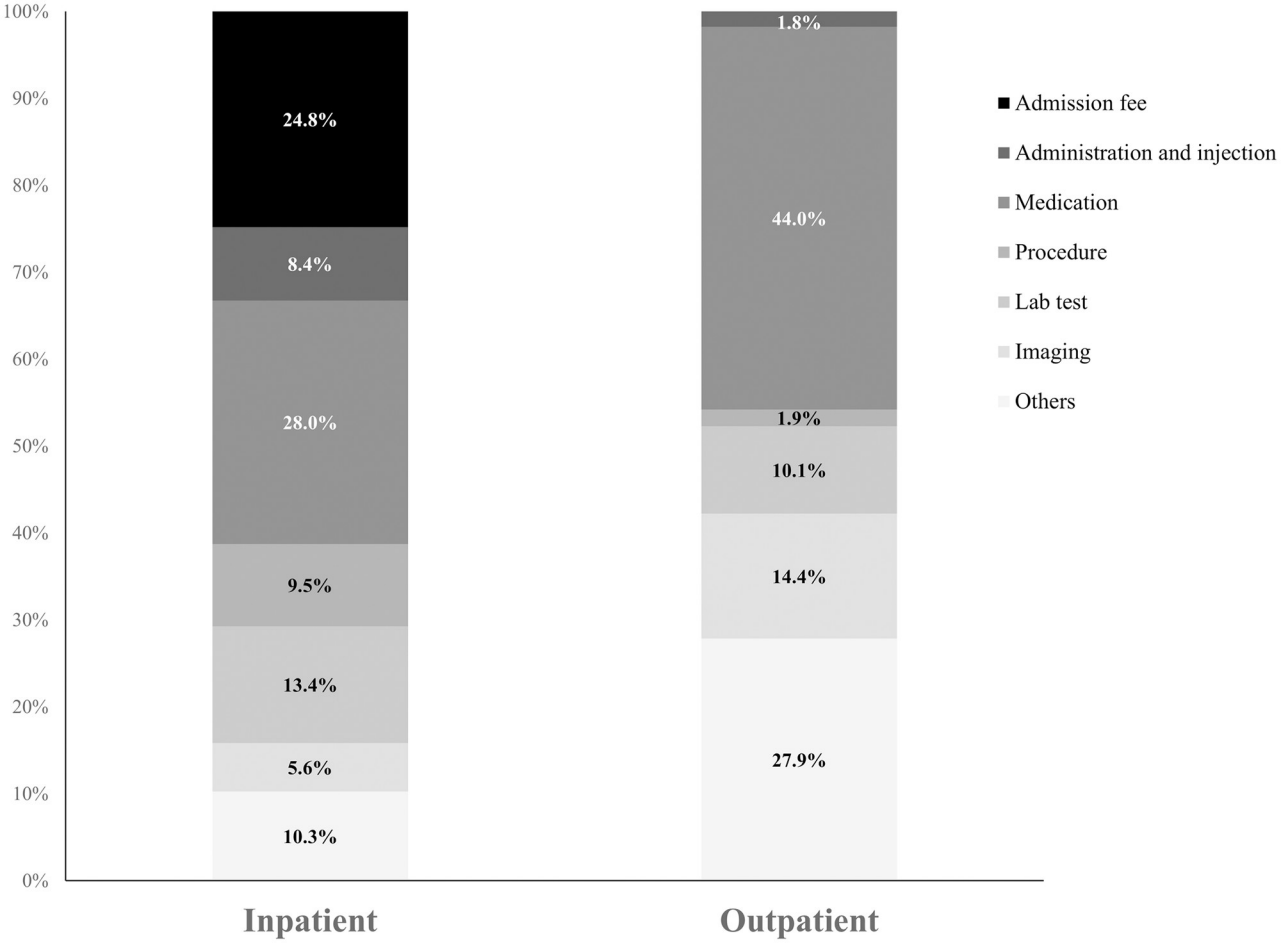
Proportions of medical costs for inpatients and outpatients during best supportive care.

The costs of admission (24.8%), medication (28.0%), and lab tests (13.4%) accounted for most of the inpatient costs, and the cost of medication (44.0%) accounted for the highest proportion of outpatient costs (Fig 2).

## Discussion

This study found that most patients with terminal cancer were hospitalized during the best supportive care period, with hospitalization days accounting for approximately 40% of their survival time. However, the proportion of ICU admissions and ED visits was not large, suggesting that few patients required urgent medical support. Patients with terminal illnesses tend to stay in the hospital because of the safe and comfortable feeling it provides, even if their stay is unnecessary [11, 20]. Not all hospitalization services were essential to these patients. A previous systematic review and meta-analysis showed that approximately 38% of these patients received non-beneficial antibiotic, cardiovascular, digestive, and endocrine treatments; and non-beneficial treatment was performed on 33–50% of patients with do-not-resuscitate orders [21]. In Korea, doctors and patients can choose clinical services more freely, and the fee-for-service method encourages excessive medical use and hospitalization [22]. According to Won et al., despite the enactment of the “Act on Hospice and Palliative Care and Decisions on Life-sustaining Treatment for Patients at the End of Life” for patients to withhold or withdraw life-sustaining treatment on February 4, 2018, in South Korea, patients still received unnecessary or non-beneficial treatment [23]. In addition, approximately 60% of the patients in this study were admitted to tertiary hospitals during best supportive care. The use of some unnecessary medical resources can also increase the medical costs of patients with terminal cancer.

For terminal patients close to death, hospice care can help relieve the discomfort of cancer and prepare them for a comfortable death [9]. Two studies have shown that aggressive and non-beneficial treatments can worsen physical distress and quality of life and increase medical costs compared to hospice care [9, 17]. Nevertheless, the systems supporting terminally ill patients, such as hospice care services, remain insufficient in Korea. In 2017, the hospice utilization rate of cancer decedents in Korea was 22.0%. There should be a system not only to examine whether medical resources are used unnecessarily but also to provide hospice care to appropriately support terminally ill patients.

For best supportive care, the average total medical cost ranged from $5,724 for colon cancer to $7,702 for pancreatic cancer, with inpatient cost accounting for a large proportion of the total cost; inpatient cost was approximately 9.4 times higher than outpatient cost. A previous study using the Korean Central Cancer Registry and Korean National Health Insurance Claims Database also showed that inpatient costs ($12,385) of cancer decedents accounted for most of the total medical costs ($15,720) in the last year before death [18]. In an Australian study, the inpatient cost (A$24,531) for patients who died from cancer was approximately 3.3 times higher than the outpatient cost (A$7,337) during the last six months of life [10]. Although previous studies similarly showed that inpatient cost was the largest contributor to the total medical cost, a direct comparison may be unavailable because of differences in healthcare systems between countries and differences in the severity of patients’ illnesses, depending on whether anticancer drugs could be administered.

This study identified several factors that affected total medical costs. The length of hospitalization had the greatest impact on total medical cost, and the period from the last anticancer drug administration to death and ICU admission increased the total medical cost. Most of the costs of lab tests and medication were incurred during hospitalization, along with the admission fee. The co-payment of patients with cancer is 5% of the total healthcare cost in South Korea. However, the medical costs during best supportive care are high and can be an economic burden to the payer as well as the patient. According to data from the Korean Statistical Information Service, a national-level database, the monthly medical cost per patient with cancer was $247 for gastric cancer, $374 for colon cancer, $592 for liver cancer, $805 for pancreatic cancer, and $712 for lung cancer [24]. The medical costs during the best supportive care were approximately 4―12 times higher than the average medical costs for patients with cancer. In addition, a previous study reported that the medical costs of patients with terminal cancer are much higher than those with other terminal diseases. To reduce the economic burden on these patients, an integrated care system should be developed to replace unnecessary hospitalization.

The characteristics and healthcare resource utilization of patients differed according to the cancer type. The best supportive care duration was the longest for gallbladder and biliary cancer, for which therapeutic agents are limited [25, 26]; whereas, it was the shortest for lung cancer, for which several available treatment drugs have been developed, even in the end-stage. Pancreatic cancer had the highest proportion of hospitalized patients, the longest period of hospitalization, and the highest medical costs. Strong analgesics should be administered to patients with pancreatic cancer because of severe abdominal pain [27]. The proportion of patients prescribed opioid analgesics was the highest in this study for pancreatic cancer. The prescription of such drugs may increase the hospitalization rate of patients with pancreatic cancer, and the total medical cost appears to increase owing to the long hospital stay.

This study has several limitations. First, because administrative claims data were used in the analysis, inaccurate codes might have introduced misclassification bias in the diagnosis of cancer. However, a previous validation study reported that the concordance rate of cancer diagnosis was approximately 90% in the Korean health insurance claims database [28], and only patients who had at least two independent claims associated with cancer were included to verify the diagnosis. Second, because claims data only contain the medical costs reimbursed by the NHIS, non-reimbursed costs were not considered in this study. More research is needed to evaluate the total economic burden on patients with terminal cancer from a societal perspective. Third, patients who died for reasons other than cancer may have also been included in this study. Because the inclusion of these patients could lead to biased results, the period from anticancer drug use to death was limited to six months to minimize the number of patients who died due to other causes.

## Conclusions

Most patients with terminal cancer are hospitalized for approximately 40% of their survival period. Hospitalization is a major driver of the total medical cost for patients with terminal cancer; thus, a system other than hospitalization is needed to support these patients clinically and financially.

## Supporting information

S1 Fig Proportions of patients with cancer who were prescribed best supportive care medication. (TIF)
