# Supplementary figures and images for: Healthcare resource utilization and medical costs in patients with terminal cancer during best supportive care
Source: PLoS One. 2022 Jun 3;17(6):e0269565. doi: 10.1371/journal.pone.0269565 (PMC9165859; doi:10.1371/journal.pone.0269565)

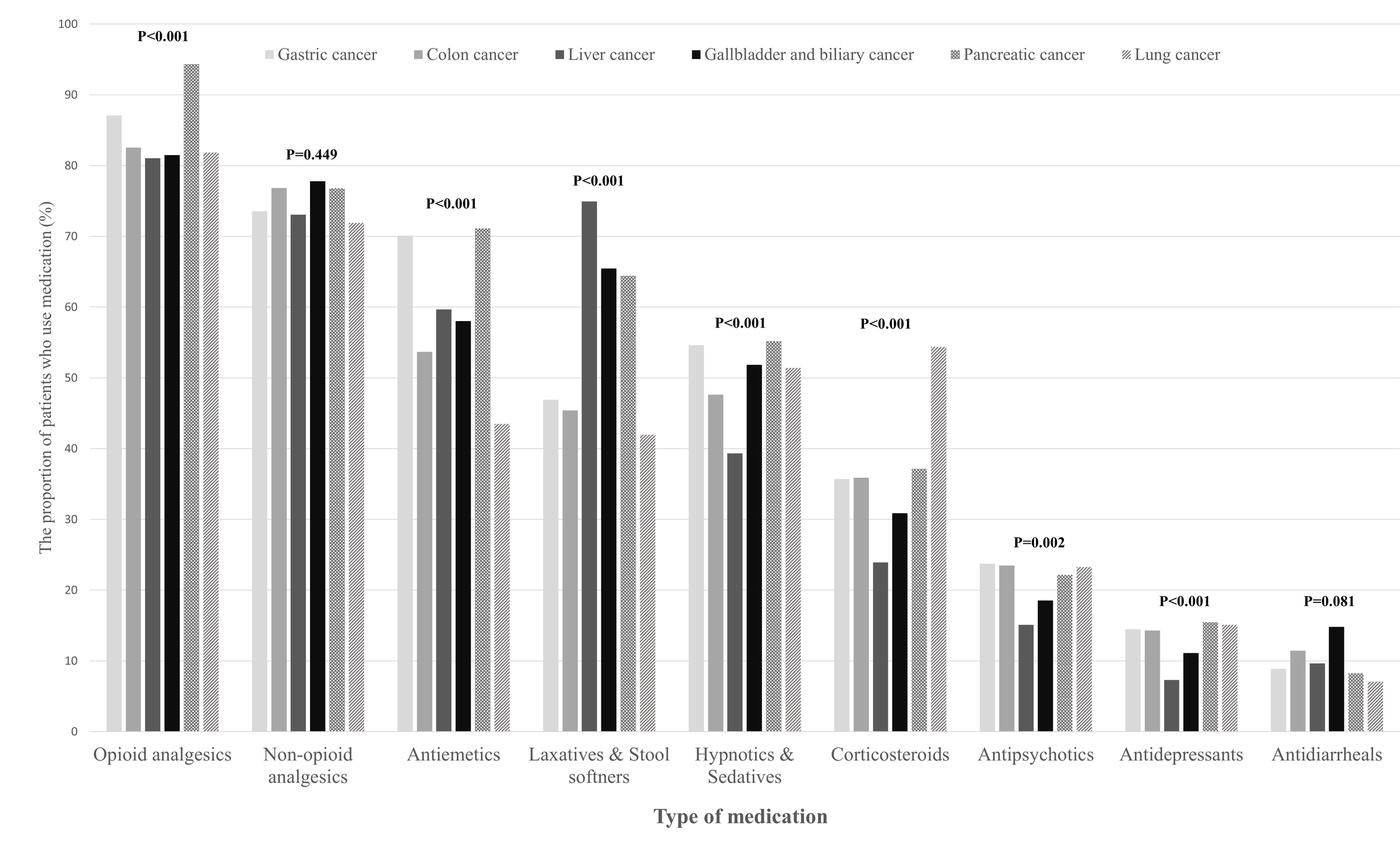

Supplement: S1 Fig — (TIF) [file pone.0269565.s001.tif]
